# Tissue Specific Electrochemical Fingerprinting

**DOI:** 10.1371/journal.pone.0049654

**Published:** 2012-11-19

**Authors:** Pavlina Sobrova, Lenka Vyslouzilova, Olga Stepankova, Marketa Ryvolova, Jiri Anyz, Libuse Trnkova, Vojtech Adam, Jaromir Hubalek, Rene Kizek

**Affiliations:** 1 Department of Chemistry and Biochemistry, Faculty of Agronomy, Mendel University in Brno, Brno, Czech Republic; 2 Department of Cybernetics, Faculty of Electrical Engineering, Czech Technical University, Prague, Czech Republic; 3 Department of Chemistry, Faculty of Science, Masaryk University, Brno, Czech Republic; 4 Central European Institute of Technology, Brno University of Technology, Brno, Czech Republic; 5 Department of Microelectronics, Faculty of Electrical Engineering and Communication, Brno University of Technology, Brno, Czech Republic; RMIT University, Australia

## Abstract

**Background:**

Proteomics and metalloproteomics are rapidly developing interdisciplinary fields providing enormous amounts of data to be classified, evaluated and interpreted. Approaches offered by bioinformatics and also by biostatistical data analysis and treatment are therefore of extreme interest. Numerous methods are now available as commercial or open source tools for data processing and modelling ready to support the analysis of various datasets. The analysis of scientific data remains a big challenge, because each new task sets its specific requirements and constraints that call for the design of a targeted data pre-processing approach.

**Methodology/Principal Findings:**

This study proposes a mathematical approach for evaluating and classifying datasets obtained by electrochemical analysis of metallothionein in rat 9 tissues (brain, heart, kidney, eye, spleen, gonad, blood, liver and femoral muscle). Tissue extracts were heated and then analysed using the differential pulse voltammetry Brdicka reaction. The voltammograms were subsequently processed. Classification models were designed making separate use of two groups of attributes, namely attributes describing local extremes, and derived attributes resulting from the *level* = 5 wavelet transform.

**Conclusions/Significance:**

On the basis of our results, we were able to construct a decision tree that makes it possible to distinguish among electrochemical analysis data resulting from measurements of all the considered tissues. In other words, we found a way to classify an unknown rat tissue based on electrochemical analysis of the metallothionein in this tissue.

## Introduction

The attention of biologists, biochemists, chemists and numerous scientists from other fields is currently targeted towards proteomics, which provides information about protein localization, structure and function, and most importantly, interactions with other proteins. Metallothioneins (MTs) were discovered by Margoshes and Valee in 1957 as newly identified proteins isolated from horse renal cortex tissue [1]. Mammalian MTs are low molecular mass proteins (app. 6 kDa) with a unique abundance of cysteine residues (more than 30% of all aminoacids) occurring in conserved sequences cys-**x**-cys, cys-**x**-**y**-cys and cys-cys, where × and **y** represent some other amino acid. Four mammalian MT isoforms (MT-1–MT-4) are known, and 13 MT-like human proteins were identified [2]. MT-1 and MT-2 are present in almost all types of soft tissues [3]. MT-3 is expressed mostly in brain tissue but also in heart, kidneys and reproductive organs [4], and the MT-4 gene has been detected in epithelial cells. The main function of MTs is metal ion transport, maintenance of oxidative-reducing conditions, and regulation of gene expression in an organism. Attention is nowadays focused on the role of MT in cancerogenesis, and on the relation of these proteins with the cancer cell cycle [5]–[11]. Although the cause and the mechanism are not clear, it is considered that its increased level is responsible for protecting cancer cells from apoptosis, and for increasing proliferation and the ability to metastasize [11].

It is not easy to detect and quantify MT due to the high content of cysteine and relatively low molecular mass. Analytical methods are usually based on a) detection of bonded metal ions, b) detection of free thiol moieties, c) protein mobility in an electrical field and d) interaction with various types of sorbent, or e) Enzyme-Linked Immuno Sorbent Assay (ELISA). However, the Brdicka reaction in connection with differential pulse voltammetry (catalytic reaction) is the only direct method able to quantify these proteins both in blood and in tissue extract samples. The differential pulse voltammetry Brdicka reaction is a sensitive and widely-used tool for determining metallothioneins –for each studied sample it generates from tens to hundreds of values of a measured signal exhibiting some relation to the composition of the sample. However, no theoretical explanation has yet been offered that could provide a complete model of the behaviour observed during the Brdicka reaction and the influence or role of MT. It is almost impossible to process all data collected during the Brdicka reaction manually, even with the help of instrument software. It is probable that each proteome has to be characterized by information resulting from a combination of several independent analytical approaches. However, finding relational dependencies is a task of such combinatorial complexity [12] that it is beyond the scope of manual evaluation to ensure an exhaustive search if the data consists of more than just a few values. Tasks of this kind can be resolved using computer processing if the size of their input is measured in dozens, but it is untreatable when the input is measured in hundreds. Since the Brdicka curve is usually described by a set of several hundred measured values, it is clear that this representation cannot be used to solve the problem of a search for relational dependencies. On the other hand, the Brdicka curve can be viewed as a smooth curve, and this kind of object can be characterized in a more abstract way, e.g. as a list of its local extremes, or using a wavelet transform. This paper reports on the results of our experiments with several abstract representations of the Brdicka curve that we tested while trying to find a method that could automatically separate biochemical samples taken from different sources, as described in Section 2.

We have focused our attention on observing the shape of the curves corresponding to different organs obtained from 28-day-old male *Wistar albino* laboratory rats, using the Brdicka reaction. The experimental data was analysed using our own software tool, which was designed and implemented with the intention to help in determining the type of tissue from which an extract was prepared. Our approach is based on several alternative descriptions of the measured curves resulting from sophisticated aggregation of the original data, leading to more compact representation by a significantly smaller set of derived attributes that maintain the strength to construct a reasonable model (in our case a decision tree) or support the intended decision. These alternatives were tested for liver, kidney, spleen, heart, brain, eye, gonads, blood and femoral muscle fingerprinting, with the intention to identify the best choice from the alternative representations of the Brdicka curve that are introduced in section 3.2.1. Our experiments reported in section 3.2.2 not only confirm that it is possible to classify with high precision an unknown tissue sample characterized only by its Brdicka curve, but also point to the importance of the neighbourhood of the inflexion point of the Brdicka curve in the interval (−1.65 V; −1.5 V). It is surprising that these inflexion points appear in a much smaller interval, namely within (−1.61 V; −1.575 V).

## Results and Discussion

### 2.1 Electrochemical measurement

#### 2.1.1 Brdicka reaction

A method for polarographic determination of proteins that contain SH-groups in an ammonia buffered cobalt(III) solution was first described by Brdicka [13]. The Brdicka reaction is a frequently employed electrochemical method for determining MT in biological samples. One of the most important results from studies of the Brdicka reaction was the discovery that, in the presence of cobalt ions in solution, even a simple sulphide ion in a concentration below the solubility product of CoS can catalyse hydrogen evolution on HMDE [14]. This confirms that the essential catalytic agent in such systems is the hydrogen atom attached to the sulphur bound to cobalt, and that whatever is bound to sulphur in addition to cobalt and hydrogen is of secondary importance [15]. In a proton nuclear magnetic resonance study of metallothionein clusters of cobalt, it was observed that the protons hydrogen-bonded to sulphur atoms binding cobalt behave anomalously in that they are unusually exchangeable, similarly to the hydrogen bonds encountered in various iron containing proteins. With hanging mercury drop electrode, the reaction of the catalytic evolution of hydrogen from slightly alkaline buffered solutions containing thiols and cobalt ions was found to take place in two potential ranges [15], [16]. The catalytic signals are a special case of the kinetic signals; the limiting catalytic current can significantly exceed the limiting current corresponding to a given catalyst concentration [17].

The experimental conditions for detecting MT using the Brdicka method were modified several times, with the aim to study the effect of the concentration of some substances in the Brdicka solution, which commonly consists of cobalt(III) complex and ammonia buffer. Raspor et al. [18] used 2 M NH_4_Cl+NH_4_OH, with 1.2 mM [Co(NH_3_)_6_]Cl_3_ and carried out the measurements within the potential range from −0.9 to −1.9 V. Olafson and Sim suggested the use of 1 M NH_4_Cl+NH_4_OH, with 0.6 mM [Co(NH_3_)_6_]Cl_3_ [19], [20]. The most frequently employed method for detecting MT using Brdicka procedures is differential pulse voltammetry. The Brdicka reaction has been used for the study of physiological concentrations of MT in many animal species [21]. The Brdicka reaction finds a wide range of use in determining MT in freshwater and sea fishes [20], [22].

#### 2.1.2 A description of voltammograms

**Figure 1:**
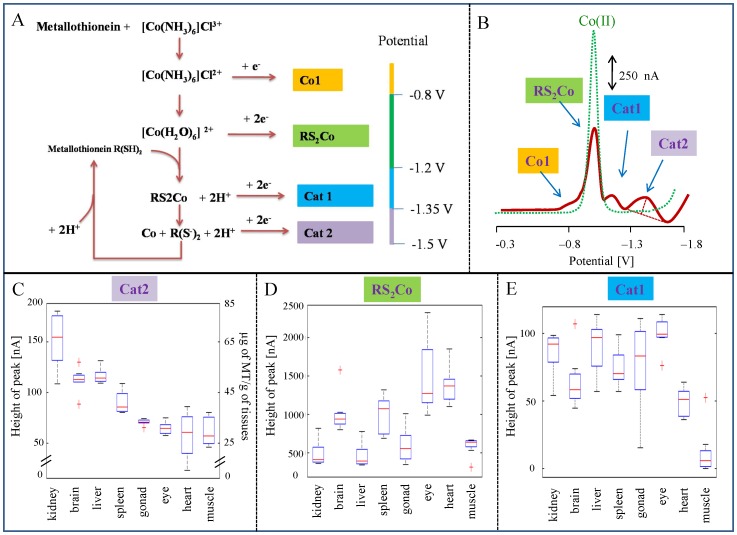
Brdicka reaction of metallothionein. (A) Presumable scheme of the sequence of electrochemical reactions at the mercury electrode when the Brdicka reaction is applied for MT analysis. (B) A typical DPV voltammogram of MT measured in the presence of a supporting electrolyte containing 1 mM Co(NH_3_)_6_Cl_3_ and 1 M NH_3_(aq)+NH_4_Cl, pH = 9.6; dotted line: voltammogram of a supporting electrolyte without MT. During MT analysis four peaks, Co1, RS_2_Co, Cat1 and Cat2 that correspond to the MT level can be observed. Heights of (C) Cat2, (D) RS_2_Co and (E) Cat1 peaks measured in extracts of various rat tissues. (C) Metallothionein level in single tissues. The highest level is in liver and kidney, i.e. organs providing detoxification, and in brain. In comparison, the level of MT in muscle and in heart is almost 50% lower.

The mechanism of the reaction is based on the catalytic evolution of hydrogen on mercury electrodes from solutions of protein-containing –SH group in ammonia buffer and hexaammincobalt chloride complex (Co(NH_3_)_6_Cl_3_), known as the Brdicka solution [15]. The mechanisms of the reaction have not been explained in detail, but it has been proposed that a complex of cobalt (II) ions with the protein, peptide or basic nitro compounds play a decisive role in the catalytic process [23]. The interaction between cobalt(II) ion and protein causes a decrease in the cobalt peak and the occurrence of two new voltammetric peaks in the potential area from −1.2 to −1.5 V (Fig. 1A). The reduction of complex R(SH)_2_ and Co(II) at potential app. −1.2 V to −1.35 V corresponds to the first catalytic signal (RS_2_Co). Two other signals, Cat1 and Cat2, correspond to the reduction of hydrogen at the mercury electrode, and can be used for quantification because their height is proportional to the concentration of MT. In addition, the signal called Co1 could occasionally result from reduction of [Co(H_2_O)_6_]^2+^ [18]. Under our conditions, we observed the formation of catalytic signals in potential from −1.2 V to −1.35 V 1.0 to 1.1 V for the first catalytic signal (RS_2_Co) and somewhere around −1.3V and −1.5V 1.2 and 1.5 for Cat1 and Cat2, respectively. Signal Co1 of varied strength was observed in potential 0.8. The hydrogen evolution mechanism at the mercury electrode in the Brdicka solution is shown in Figure 1B.

#### 2.1.3 Determining the metallothionein content in rat tissues

Using electrochemical detection of metallothionein, we monitored its content in the blood and in rat body tissues: liver, kidney, spleen, heart, brain, eye, gonads, blood and femoral muscle (number of different tissues taken into consideration p = 9). The catalytic peak Cat2 was used to quantify metallothionein (Fig. 1C). A comparison of the average MT levels in single tissues shows their variability (Fig. 1C). The highest content was found in kidney (67.0±1.1 µg/g) and liver (48.7±0.9 µg/g), i.e. in organs responsible for detoxifying xenobiotics. The content was two times higher than in other organs. A higher level was also determined in the brain (50.5±1.5 µg/g) and in the spleen (41.5±1.4 µg/g). The concentration in the brain corresponds only to the content of isoform MT-3, which occurs in the brain only [24]. The MT level in the heart (without blood) is near to the MT level in muscle, which can be explained by the similar physiological function of the two tissues. Moreover, we made a statistical evaluation of the differences in the MT content determined in the organs. We found that the MT content in the heart was not statistically different from the MT content in the gonad and in the eye. In addition, the MT content in the gonad was not statistically different from the MT content in the eye. All other mutual differences were significantly different (p<0.05). In addition to the height of peak Cat2, the heights of RS_2_Co and Cat1 were determined and are shown in Figures 1D and 1E, respectively. However, no dependence on concentration or on other parameters, including type of tissue, was observed.

In addition, we focused our attention on the shape of the curves corresponding to the different organs. We found that each tissue provided a voltammogram of characteristic shape. The curves differed not only in their height and in the potential of the peaks, but also in their shape. Some voltammograms contained RS_2_Co, Cat1 and Cat2 only, but in other curves, peak Co1 was detected at −0.7 V. Voltammograms with three peaks (without Co1) were obtained by measuring the spleen, gonad and muscle homogenates. Analyses of kidney, liver, brain, eye and heart homogenates gave four peaks. Although apparent differences among measured voltammograms were observed visually, we were not able to distinguish between voltammograms of certain tissues using standard data treatment tools.

### 2.2 Mathematical data analysis, and some of its implications

#### 2.2.1 Alternative data set representations

In our case study, each Brdicka curve represented a unique measurement. Since each sample was measured 5 times, our dataset consisted of 40 curves (5 measurements for each of 8 rats) for each body part. The main goal of our data analysis was to find a preprocessing method that would lead to the definition of a small number of derived attributes that.

- characterize the voltammograms well enough to allow the construction of a classification model for identifying the corresponding body parts from the measured data curve, and
- permit a natural interpretation in the electrochemical domain.

The wavelet transform, described later in this section, proved to offer efficient means for constructing a set of derived attributes of this kind. Using these attributes resulting from careful processing of the original data curves, we were able to specify interesting parts of curves exhibiting the most significant differences for various body parts, and to design decision trees that distinguished different body parts.

The first step in our analysis was data cleaning. The data set contained 5 data curves for each sample (body part of one of the rats) resulting from 5 repeated consecutive measurements of the identical sample. Obviously, it was expected that the 5 curves resulting from repeated analysis of a single sample should be very similar to each other. Visual inspection allowed us to point to 8 cases where we identified one of the five curves under consideration that was dramatically different from the others. There seemed to be no reason for this behaviour except a measurement error – errors cannot be excluded since the analytical process is highly automated: there are numerous possible sources of errors, e.g. failure in dosing the sample or in the basic electrolyte, or the occurrence of an impurity somewhere in the equipment. A situation of this kind occurred in samples from rat no. 6 and rat no. 8 taken from muscle, blood, heart or gonad. We carefully assessed the corresponding data, and we came to the conclusion that these 8 measurements out of a total of 360 must be the result of a failure during the measurement (e.g. 2 of the 8 measurements proved to be incomplete). We therefore decided to neglect these measurements, and to delete these dramatically different curves from the original data set. The resulting dataset (used for our experiments – described in the text below) therefore consisted of 352 Brdicka curves only, because 6 out of 72 groups of repeated measurements did not include 5 curves, as originally planned, but a lower number (usually 3 or 4).

There is a natural representation of the continuous signal that is close to the representation used in biochemistry, namely an array of the positions and functional values of its local extremes (local maxima and minima). The main advantage of this approach is that the accurate position of all the extremes is maintained, including the corresponding functional value.

The preliminary review of the voltammograms showed that almost all the curves had six local extremes. The only exception occurred in the graphs depicting data obtained from samples of blood and muscle - no more than 4 extremes were observed here. The positions of the extremes (i.e. their horizontal coordinates) seemed to be very similar for the same body part, while they showed significant variability among different body parts. Due to this inter-part variability, it was impossible to specify for all samples a single short common interval with firm ranges where one should search for the first (or other) extreme. Consequently, the full range of values had to be carefully scanned to identify reliable positions of all local minima and maxima.

Since the treated data represents a discrete signal, it was not possible to identify the extremes as those points where the derivation is equal to 0. Instead, we had to rely on an appropriate approximation of a discrete signal derivation, which can be obtained using a simple differentiator, namely a first order FIR filter described by the a differential equation below.
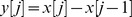



After applying this differentiator to the original signal, we searched for zero crossings of this new signal, because these points correspond to the exact position of the extremes of our interest. The zero crossings were obtained as an array of logical values resulting from normalization of the differential signal, using a function similar to ‘signum’ (this function assigns value+0.5 to input higher or equal to zero, and value −0.5 elsewhere). By applying the differentiator to the normalized signal and setting up the length of the new signal by adding zeros, we obtained a binary signal with two possible values, namely zero and one, of the same length as the original signal. The values of the local extremes were obtained by indexing the original signal with our computed signal of logical values.

A straightforward application of the suggested simple algorithm for identifying the local extremes proved to have a serious drawback due to the experimental nature of the signals. Some of the signals were not entirely smooth, and consequently some minor waves appeared in the graph, resulting in the identification of insignificant extremes within a small interval. These minor local extremes can be caused by noise, and are difficult to recognize and remove. Luckily, this problem occurred in just a few cases, which could be eliminated by setting the requirement for a minimum distance between two neighbouring extremes, which were merged if their distance was below a predefined threshold. To do this, first the distance between all neighbouring extremes was calculated by applying a differentiator to the positions of the extremes, and the places below the threshold were identified as the positions of the suspicious pairs of extremes – for each suspicious pair, one of the two was removed. This simple method is far from optimal, and should be improved or replaced by a better method later. Its main advantage is low approximate time complexity – it is linearly dependent on the number ***d*** of treated values, i.e. O (***d***) = ***d***.

To ensure uniformity of the data representation during further processing of the local extremes of the Brdicka curves, and their utilization in the design of a decision tree, we decided to focus our attention first on signals having precisely 6 significant extremes. This requirement was not met by the blood and muscle samples, which were excluded from the initial part of our experimental evaluations. In the rest of our paper, whenever our attention is restricted to a smaller set of Brdicka curves, we will offer a clear description of how they were selected (e.g. the data without all the blood and muscle samples).

Each curve from our data set is described by 518 points obtained from equidistant measurements in the interval (−1.8 V, −0.7 V) – domain experts confirmed that it is safe to take the line connecting all these points as a reasonable approximation of a continuous line. A well-known approach for handling data of this type is to apply Haar’s Simple Wavelet (HSW) transform [25], [26], [27], which significantly compresses the input data by approximating the continuous line by a stepwise function with 2*^k^* steps (columns) of the same width, where parameter *k* is referred to as the HSW level. To ensure this plan, HSW transformation sets a strict requirement on the number ***d*** of points used for representing the curve - this number has to be a power of 2. In this case, log ***d*** is the upper limit for the HSW level parameter denoted as *level_max_*. The nearest power of 2 to the number of points that we have, i.e. 518, is 512 = 2^9^. In order to use HSW for our Brdicka curve data, we set *level_max_* = 9, and we were forced to abandon six points only. For that purpose, we decided to select the 6 points with the value on the horizontal axis close to (−0.7 V), because their measured values were the same for most of the curves.

Haar’s Simple Wavelet transform has a single parameter referred to as *level*, which cannot exceed *level_max_* and which specifies the number of iterations of the process described below, as well as the number of coefficients to be used for a description of the curve. More precisely, the number of coefficients is equal to 2*^level^*. The initial wavelet coefficient *c*0 represents the mean of the whole curve. In the next steps the following process is repeated: the domain is divided into two equal parts and each part is analysed separately (the mean of this part is considered) and is then compared to its ‘parent part’ to obtain further wavelet parameters. The interpretation of the resulting wavelet coefficients is not very transparent. We therefore applied the inverse wavelet transform, which results in the same number of novel derived attributes (denoted as *coef*0, …, *coef*15 in the case that *level* is set to 4) computed by aggregating the original wavelet coefficients. These new attributes offer a more natural description of the original curve, because they represent its approximation by a stepwise function with 2*^level^* steps of equal width which roughly copies the shape of the original data: each new attribute *coef*0, …, *coef*15 corresponds to the average of the original curve in the considered interval (step).

**Figure 2:**
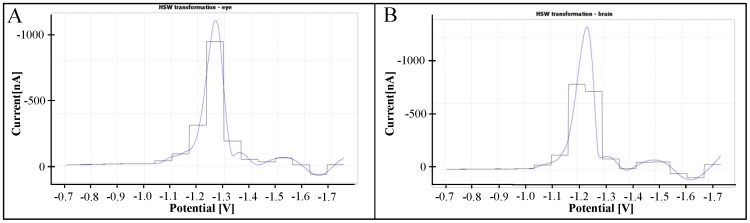
Transformation of DP voltammograms. (A) Haar’s Simple Wavelet transformation – brain. (B) Haar’s Simple Wavelet transformation – eye.

Initially, we started with parameter *level* = 4, resulting in the output set of all wavelet coefficients with 16 members. In other words, the transformation that was used took the original 512 measurements (points of the curve) from the input dataset and transformed them into 16 derived attributes that can be visualized as the height of 16 steps of constant width, see Figure 2A. This transformation ensuring significant compression of the input data proved to be able to characterize the original data reasonably well as long as each peak of the original curve appears inside one of the steps, see Figure 2A, where the values of *coef*0, …, *coef*15 (corresponding to the average value of the function within the considered interval or step) roughly copy the shape of the original curve. However, a steep peak on the break between two steps can influence the values of the derived coefficients in such a way that these values become misleading. A situation of this kind can be observed in Figure 2B in the vicinity of −1.25 V, where the value of the corresponding derived attributes no longer copies the shape of the original data well enough.

The simplest way to deal with the problem of the steep jump would be to use finer steps in its neighbourhood. However, all the steps in the simple wavelet transform have to be of the same width. In our case, the constant width requirement suggested an increase in the parameter level of the HSW transform from *level* = 4 to *level* = 5. This parameter change provided a better description of the Brdicka curve around the steep jump approximately in the middle of the curve. Setting the *level* to 5 resulted in the creation of 32 new derived attributes that could be used for constructing a decision tree in the next section. To ensure all the data preprocessing steps described here, we developed a dedicated SW tool implemented in the JAVA environment that also supports visualization of the data.

#### 2.2.2 Classification

Classification models were designed using two groups of attributes separately - attributes describing local extremes (see section 3.1.3), and derived attributes resulting from the *level* = 5 wavelet transform (see section 3.1.3). Among various available classification models [28], we selected decision trees because they highlight possible dependencies among the considered attributes, and thus they offer a clear insight into the task, due to the algorithm used for constructing the decision tree. This algorithm applies iteratively a routine for identifying the most informed attribute to the considered dataset. This attribute is then used for partitioning the dataset – further on, a significantly smaller dataset is processed and in this way the complexity of the task is reduced step by step. Those attributes that appear in the names of the upper nodes of the decision tree (close to its root) seem to be of special importance - the decision tree can also be understood as a feature selection algorithm.

The Rapid Miner software tool and its Decision Tree module were applied [25]. The parameter named *minimal leaf size*, corresponding to the minimal number of instances per leaf was set to 10 to prevent over-specialization when the leaves of the decision tree could be characterized by the attribute values of a single rat (one rat = 5 curves). This setting helped in finding more general decision trees. In both experiments described in the following two paragraphs, which used two different groups of attributes selected for an abstract representation of the Brdicka curve, we applied the same approach for estimating the quality of the designed model, namely 10-fold cross-validation. The available data *S*, corresponding to different Brdicka curves, was divided into 10 disjunctive sets *S*_1_,…, *S*_10_, each of which maintained the same percentage of the considered body tissues as the original set (stratified samples). For *i* = 1 to 10, the following experiments were performed: the decision tree model was created from the training data set (*S* - *S_i_* ), and was tested on the remaining data, namely on *S_i_*. The overall results of the 10 experiments were summarized using a confusion matrix with columns denoted by the body part from which the sample was taken, and with rows denoted by the classification suggested by the decision trees (Table 1). Consequently, the resulting table denoted as a confusion matrix for the experiment depicts all the correctly classified examples on the diagonal of the matrix, while all the other points represent errors. Moreover, this presentation of the results enables us easily to identify the most frequent mistakes or confusions appearing in our data, namely the names of the body tissues that the misclassified examples came from, and the predicted class that does not seem to be fully reliable.

**Table 1:** Confusion matrix for decision trees constructed from attributes obtained by a wavelet transform of level 5.

	true liver	true blood	true kidney	true brain	true eye	true spleen	true heart	true muscle	true gonad	Class precision
**pred. liver**	**40**	0	0	0	0	0	0	0	0	100%
**pred. blood**	0	**39**	1	0	0	0	0	0	0	97.50%
**pred. kidney**	0	0	**37**	1	0	0	0	0	0	97.37%
**pred. brain**	0	0	2	**34**	0	0	0	0	0	91.89%
**pred. eye**	0	0	0	1	**39**	0	1	0	0	95.12%
**pred. spleen**	0	0	0	0	0	**38**	0	0	0	100%
**pred. heart**	0	0	0	4	1	0	**37**	0	0	89.10%
**pred. muscle**	0	0	0	0	0	1	0	**38**	0	97.44%
**pred. gonad**	0	0	0	0	0	0	0	0	**37**	100%
**Class recall**	100%	100%	92.50%	85.00%	97.50%	97.44%	97.37%	100%	97.37%	

Our first experiment was inspired by common notation used in biochemistry. To ensure uniformity of the data representation during further processing of the local extremes of the Brdicka curves, and their utilization in the design of a decision tree, we decided to focus our attention on signals with precisely 6 significant extremes. There are only two body parts that do not meet this requirement, i.e. the blood and muscle samples. The corresponding samples were therefore neglected. What remained was the set of 280 voltammograms resulting from 5 measurements of samples taken from 7 different body parts from 8 rats. The cleaning described above reduced this number further to 275 curves. First, a decision tree was constructed from the coordinates of the well-known points RS_2_Co, Cat1 and Cat2, only. The confusion matrix of the resulting decision tree exhibited many errors in the classification of the body part, and the accuracy obtained during 10-fold cross-validation was 80.77% ±5.01%. It is worth noting that the body parts were assigned to three groups on the basis of the branches on which they appeared: the first group comprised liver, kidney and gonad, the second group was brain and heart. while the last group was eye and spleen. Surprisingly, most of the errors in the confusion matrix misclassified one body part for another in the same group, or in other words misclassification did not occur between two different groups.

In the second experiment with the same data set of 275 curves, the attributes from the first experiment were complemented by additional local extremes appearing in the studied interval (−1.8 V, −0.7 V), i.e. by the local maxima of the considered voltammograms. It should be stressed that this is a major enhancement of the former data representation, since points RS_2_Co, Cat1 and Cat2 are the local minima from a purely mathematical point of view, though they look like maxima. This is due to the standard representation of biochemical graphs, where the two axes are mirrored. The confusion matrix of decision trees constructed from all 6 local extremes proved to have a somewhat lower number of errors, but it still failed to classify gonad, spleen and brain correctly. The accuracy obtained during 10-fold cross-validation rose to 86.56% ±4.54%. While the resulting decision trees were simpler, they pointed to the same three groups of body parts as before.

**Figure 3:**
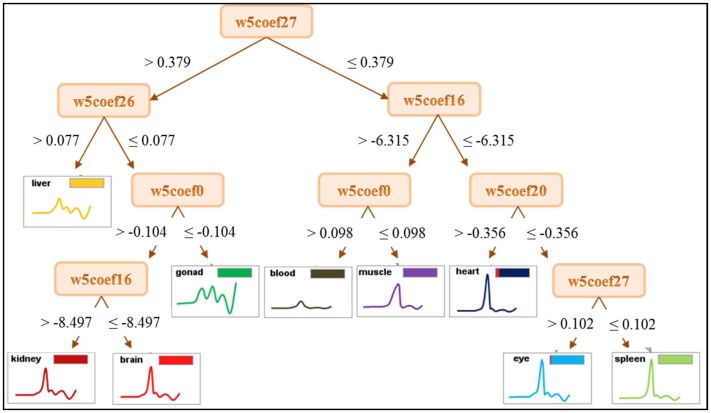
Decision tree of all samples from the cleaned dataset.

In the last classification experiment, decision trees were constructed from 32 derived attributes taken from wavelet transformation, see Figure 3. This meant that we were no longer forced to skip data from blood and muscle measurements, because wavelets provide a good description even of curves with a lower number of extremes. Thus we could take advantage of all the data obtained after cleaning the original dataset, and again work with the full set of 352 curves. Although this makes the classification task more complex, since the data has to be classified into 9 classes (as against 7 before), the resulting classification is nearly perfect: 10-fold cross-validation reported impressive accuracy of 96,31% ±2.56%. The only body part exhibiting serious error is the brain (Table 1).

The decision tree that was created divided the Brdicka curves into 3 clusters again: the first group containing liver, kidney, gonad and brain, the second group containing blood and muscle, and the last group containing heart, eye and spleen. The three groups identified earlier were then merged into two groups: the group with liver, kidney and gonad curves was supplemented by brain curves, while the heart curves were added into the group with eye and spleen curves. We can observe a certain correspondence between this clustering and the MT levels of the body parts (Fig. 1C). MT levels only are not sufficient to provide a reasonable explanation. In the case of the tree based on wavelet coefficients, we cannot neglect the importance of attributes *w*5*coef*27 and *w*5*coef*26. They correspond to the part of the curve between the point denoted as Cat2 and the last local maximum, denoted as Max3. The corresponding part of the Brdicka curves is explored in the next section.

#### 2.2.3 Analysis of the interval between point Cat2 and local extreme Max3

Our aim was to gain a better understanding of the behaviour of the Brdicka curves in the neighbourhood of those selected parts that are described by attributes *w*5*coef*26 and *w*5*coef*27. First, we tried to find whether there is some relation between the values of these attributes and attributes w5coef24 and w5coef25. Very preliminary information can be obtained by visualizing these points. At first sight this may seem to be a bizarre goal, as human imagination is limited to 3 dimensions. Fortunately, we can use the RadViz visualization solution briefly explained below to depict relations in data with more than three dimensions [29], [30], [31].

RadViz (Radial Coordinate Visualization) [29], [32] is a visualization method that uses the Hookes law from physics for mapping a set of *n-*dimensional points into a plane. It offers a unique method which can help to identify relations among data. Its main advantage is that it needs no multiple projections, while it provides a global view on multidimensional data.

Each RadViz mapping of points from *n*-dimensional space into a plane is uniquely defined by the position of the corresponding *n* anchors (points S*_j_*), which are placed in a single plane. The anchors are most often situated around a circle. In this case, each anchor is characterized just by its angle α*_j_*, which specifies the radial distance of this anchor *j* from the position of the first anchor. The point y = [*y*_1_, …, *y_n_*] in an *n*-dimensional space is mapped to the point *u* = [*u*_1_, *u*_2_] with the following coordinates:
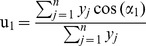


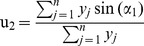



**Figure 4:**
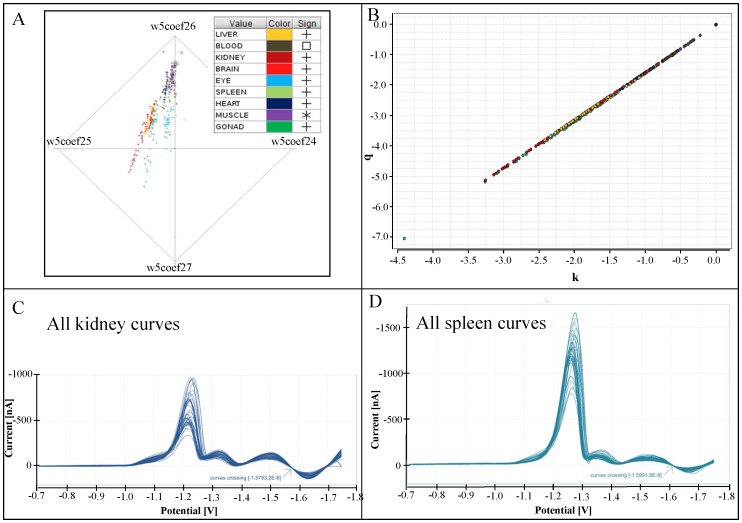
Similarities in DP voltammograms. (A) RadViz image for the *w5coef24 … w5coef27* projection of selected wavelet coefficients. (B) Approximation of the Brdicka curve between points Cat2 and Max3 by the line *y = kx+q*. (C) Cross point for the set of kidney and (D) spleen.

Figure 4A shows the resulting RadViz image for the *w*5*coef*24 … *w*5*coef*27 projection of the selected wavelet coefficients: each curve is represented by 4 values of its coefficients *w*5*coef*24 … *w*5*coef*27, and it is depicted by a unique point. Surprisingly, individual body parts seem to form lines in this image – unfortunately the image with all classes is confusing in black and white colour. To make our argument clearer, we decided to limit the number of body parts presented in this picture. The linear dependence among values *w*5*coef*24, …, *w*5*coef*27 could be explained by the existence of a significant linear section in the considered part of the Brdicka curves, i.e. between point Cat2 and local extreme Max3 (it is the part of the curve between approximately −1.5 and −1.6 V on the horizontal axis).

The next step in our analysis is an approximation of the Brdicka curve between points Cat2 and Max3 by a line *y* = *kx*+*q*. The best point at which the line can be anchored is the inflection point. We proceeded as follows for each curve: 1) The inflection point of the curve was found. 2) The nearest neighbourhood of the inflection point was selected (5 points to the left and to the right). 3) Line coefficients *k* and *q* were counted using well-known linear regression formulas, where number of points *m* = 11.
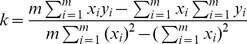


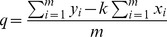



Now, each Brdicka curve in the considered interval is characterized by a pair of coefficients *k* and *q* – their values can be depicted in a plane, see Figure 4B. The resulting relation is almost perfectly linear – a closer inspection shows that there is a narrow bundle of lines corresponding to the individual body parts. Does the existence of linear dependence between the values of the calculated coefficients *k* and *q* characterizing our curves imply something we can observe in the original images of these curves? Yes, it means that all the considered lines cross at a single point, the coordinates of which can easily be calculated, as we show briefly:

Let us restrict our attention to the considered linear approximations *y* = *kx*+*q* of all the curves corresponding to a selected unique body tissue. Moreover, let us suppose that the value of *q* is a linear function of *k* with the following coefficients α; β, i.e. it holds that *q* = α*k*+β. Assuming this relation, we can substitute for *q* in the equation *y* = *kx*+*q* of the original line between the points Cat2 and Max3 in order to obtain *y* = *kx*+α*k*+β or better to obtain *y* = *k* * (*x*+α)+β. Now, it is clear that all the considered lines share exactly the point <−α; β>. Since we assume that our curves in this part of the voltammogram are well approximated by the considered lines, the Brdicka curves corresponding to a single body part should also cross at a single point. Namely, they have to share the point <−α; β>.

**Figure 5:**
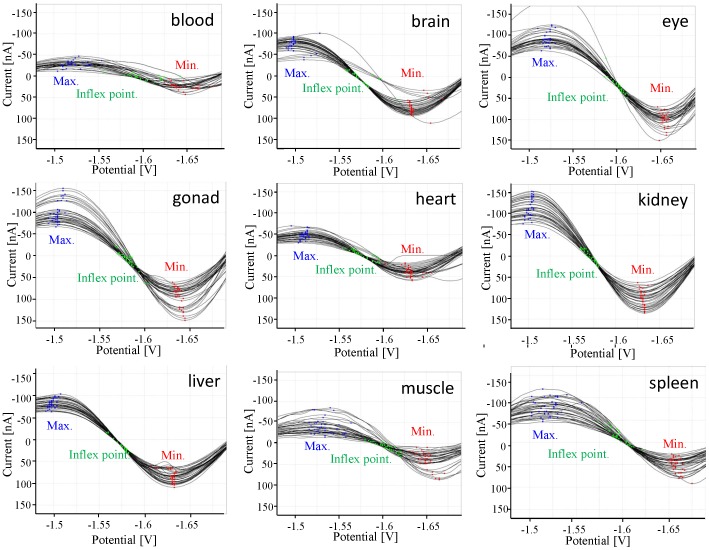
Detail of intersections of Brdicka curves for all considered body tissues.

Figure 4C and 4D confirm this hypothesis by showing the cross points for all the voltammograms obtained for kidney and spleen. Figure 5 depicts in detail the relevant part of the Brdicka curves for all the studied tissues. The intersection points are not identical for different issues, which match the fact that the calculated values of coefficients *k* and *q*, as depicted in the Figure 4B, form not a single line but a very narrow bundle of lines (one line for each body part) similarly as in the RadViz visualization. Each body part is therefore characterized by its own values α and β. The correlation coefficient between -α and the concentration of MTs in tissue is *r* = 72%.

The surprising finding that there is a single common point at which all the Brdicka curves intersect (Figs. 4C and 4D), resulting from the same tissue of various rats described in the previous paragraph, made us ask the final question addressed in this paper: Is there some precise mathematical characterization of this common point? Could this point be positioned close to the inflection point that we calculated at the beginning of the previous paragraph? Figure 5, which shows the local extremes as well as the inflection points for each curve, hints at the answer to this question. We can confirm that the intersection point is in the vicinity of the inflection point. Unfortunately, this image also proves that neither the position of the inflection point nor the position of the intersection point is sufficient to support the classification of an as yet unclassified Brdicka curve. The details in Figure 5 highlight, for example, that the part of the Brdicka curves considered for liver, kidney, brain and heart are very similar in the interval <−1.7, −1.45>: in order to distinguish among them, more information would be needed about the measured values outside this interval. A description based on the wavelet coefficients presented in section 3.2.1 seems to suggest a promising direction for providing a compact, expressive and valuable representation of the Brdicka curves treated here.

### 2.3 Biological importance of the discovered phenomenon

**Figure 6:**
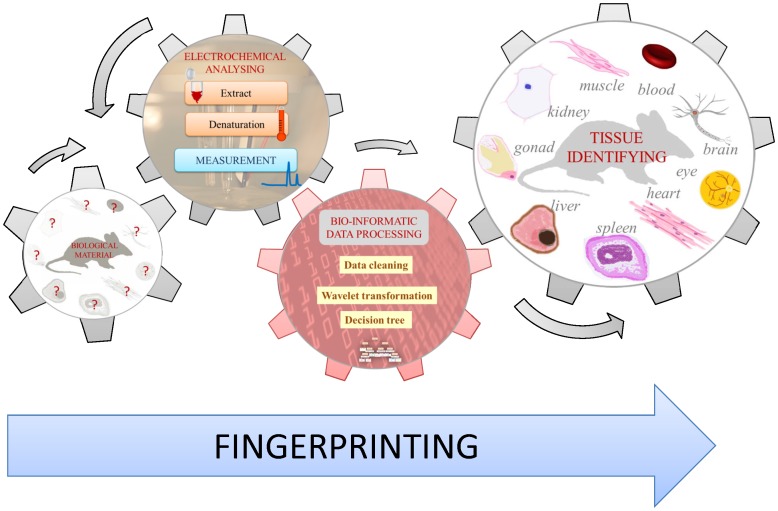
Suggested Tissue Electrochemical Fingerprinting is based on sampling a tissue, which is then extracted and heat treated. The denatured sample is further electrochemically analysed using the differential pulse voltammetry Brdicka reaction. The data that is obtained is processed using an optimized protocol, and a tissue is identified.

Tissues differ between themselves from the numerous points of view including the most basics as type of cells. The morphology as well as biochemistry of the cell are various among tissues. Distinguishing of tissues is easy, when fundamental microscopic evaluation is done. If we consider possibility of distinguishing between tissue extracts prepared using various preparation protocols, we are not able to determine the tissue, in spite of the fact that there are great biochemical differences. In our knowledge, there is no easy-to-use and low cost method how to distinguish tissues based on their extract analysis. Based on the scheme shown in Figure 6, coupling of DPV Brdicka reaction with above optimized software is able to distinguish liver, kidney, spleen, heart, brain, eye, gonads, blood and femoral muscle extracts with confidence above 95%. Considering number of samples and number of replicates, the confidence is one the bests, which can be achieved and could be of great interest for metabolomics, which can be seen as data driven strategy trying to find markers of a situation under study without a priori hypothesis. These types of studies has rapidly caught the attention and evolved from the simple pattern recognition strategy, which was a great innovation at its origins, to the interest for the final identification of markers responsible for class separation, i.e., from data to knowledge [33].

One may speculate that the suggested approach may reveal some interesting biochemical features of a sample. Considering the fact of differences of tissues, Brdicka reaction can somewhat determine compounds, of which level is strictly dependent on a tissue type. Rudolf Brdicka, which was born one hundred years ago, published in Nature in 1937 his discoveries about using polarography to diagnose a tumour disease [34], [35]. He found out a sensitive polarographic “protein effect”, conspicuously exhibited by serum, which he explained as due to the catalytic activity of the sulphydryl groups of proteins. The “protein effect” consisting of the appearance of characteristic wave on the current voltage curve, has been always found larger in normal serum sample than when the same procedure with cancer serum was used [34], [35]. One year ago, Brdicka’s colleague Jaroslav Heyrovsky, holder of Nobel Prize in Chemistry 1959, published a paper in the same journal, where he summarized results obtained in the field of Polarographic Research on Cancer [36]. Heyrovsky believed that this field of study would be of general interest of many scientific groups around the world. But he was mistaken. Since then, electrochemistry has been slowly disappearing from tumour disease diagnostics due to using modern techniques of analytical chemistry and molecular biology. Thus, this unique and interesting technique has not been used with several exceptions [19] for more than fifty years. Based on the above mentioned facts Brdicka discovered very similar behaviour, i.e. difference between two types of biochemically difference extracts. The possible explanation of this phenomenon probably lies in the presence of some proteins, mainly metallothioneins and heat shock proteins, with the ability to withstand heat treatment.

Metallothionein as low molecular mass thiols are abundant in all tissues but at low concentrations. Due to the presence of four isoforms, there are also differences in the concentrations of these isoforms with some remarkable exceptions as the presence of MT-3 in brain only. Considering the fact that differential pulse voltammetry Brdicka reaction belongs to the method mostly sensitive to MTs, differences of the content of individual isoforms should be one of the main reasons for variances in the voltammograms. Heat shock proteins (HSPs) are ubiquitous proteins in eukaryotic cells. As the name suggests, HSPs are induced in cells exposed to heat although stress proteins (Chaperonins) can be induced by many different kinds of insults allowing the cells to survive in otherwise lethal conditions [37]. Mammalian HSPs have been classified into six families according to their molecular size: HSP100, HSP90, HSP70, HSP60, HSP40 and small HSPs (15 to 30 kDa) including HSP27 [38], [39], [40]. High molecular weight HSPs are ATP-dependent chaperones, while small HSPs act in an ATP-independent fashion [41]. Each family of HSPs is composed of members expressed either constitutively or regulated inductively and is targeted to different subcellular compartments [42]. Constitutive activation of HSPs may occur in cells even in the absence of environmental stressors. HSP-90 can constitute up to 1% of total cellular protein in unstressed cells indicating that these proteins have a role in maintaining protein conformation even under normal conditions [38]. Based on the constitutive expression and relative constant level of these proteins in tissues, these can be also considered as the main contributors to tissue specific phenomena determined by DP voltammograms. Due to differences in physico-chemical properties and concentrations of the metabolites, but also due to differences in matrix properties, DPV Brdicka reaction was proving to be capable of giving us the information on tissue specificity and also, we can assume that some other biologically interesting phenomena including cancer should be also detected.

## Materials and Methods

### 3.1 Chemicals

Rabbit liver MT (MW 7143 g/mol), containing 5.9% Cd and 0.5% Zn, was purchased from Sigma Aldrich (St. Louis, USA). Co(NH_3_)_6_Cl_3_, and the other chemicals were purchased from Sigma Aldrich (Sigma-Aldrich, USA) unless noted otherwise. A stock standard solution of MT (10 µg mL^−1^) was prepared with ACS water (Sigma-Aldrich, USA) and stored in the dark at −20°C. Working standard solutions were prepared daily by diluting the stock solutions with ACS water. The pH value was measured using a WTW inoLab pH meter (Weilheim, Germany).

### 3.2 Animals

#### Ethics statement

The research was approved by the Independent Ethics Committee at Mendel University, Brno, Czech Republic. Selected 28-day-old male *Wistar albino* laboratory rats were used without any treatment in our experiments. Eight experimental animals were used. They were kept in a *vivarium* with a controlled air temperature (23±1°C) and photo-period (12 hours day: 12 hours night with maximal intensity 10,800 LUX). Tempered feed mixtures of natural barley and drinking water were accessible *ad libitum* for four weeks. At the end of the experiment, the animals were euthanized and the tissues and blood were sampled.

### 3.3 Preparation of the Biological Sample

Rat tissues (liver, kidney, spleen, heart, brain, eye, gonads and femoral muscle) and rat blood were used for the analysis. The animal tissues were mixed with extraction buffer (100 mM sodium phosphate, pH 6.8) and subsequently homogenized using a semi-automatic homogeniser (Schuett homgen, Schuett-Biotec, Germany). The homogenates and also the blood samples were centrifuged at 10,000 g for 15 min at 4°C (Eppendorf 5402, USA). Further, the samples were heat treated at 99°C in a thermomixer (Eppendorf Thermomixer Comfort, USA) for 15 min. with occasional stirring, and then cooled to 4°C. The denatured homogenates were centrifuged at 4°C, 15,000 g for 30 min. (Eppendorf 5402, USA). Heat treatment effectively denatures and removes high molecular weight proteins out from samples [18], [43], [44]. The supernatants that were obtained were 100 × diluted with extraction buffer (100 mM potassium phosphate, pH 6.8) prior to the electrochemical measurements.

### 3.4 Electrochemical Determination of Metallothionein

Electrochemical measurements were performed with a 747 VA Stand instrument connected to a 746 VA Trace Analyzer and a 695 Autosampler (Metrohm, Switzerland), using a standard cell with three electrodes and a cooled sample holder (4°C). A hanging mercury drop electrode (HMDE) with a drop area of 0.4 mm^2^ was the working electrode. An Ag/AgCl/3M KCl electrode was the reference, and a glassy carbon electrode was the auxiliary electrode. GPES 4.9 supplied by EcoChemie was employed. The Brdicka supporting electrolyte containing 1 mM Co(NH_3_)_6_Cl_3_ and 1 M ammonia buffer (NH_3_(*aq*)+NH_4_Cl, pH = 9.6) was used, and was changed after each analysis. The DPV parameters were as follows: initial potential of −0.7 V, end potential of −1.75 V, modulation time 0.057 s, time interval 0.2 s, step potential 2 mV, modulation amplitude −250 mV, E_ads_ = 0 V. All experiments were carried out at a temperature of 4°C (Julabo F12 cooler, Germany).

### 3.5 Data Resulting from the Measurements and Our SW Tool for Processing the Data

**Figure 7:**
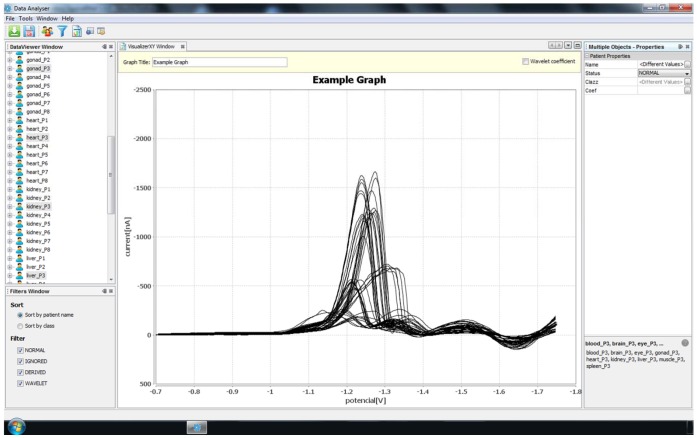
A screenshot of the SW tool that was used, depicting all Brdicka curves measured for all the body parts of a single subject, namely P3.

Our software tool was specially created for the tasks described in this paper. The whole system is built in the Netbeans platform framework for Java desktop applications, using an integrated development environment (IDE) for developing with Java. Our tool is structured into four main modules that carry out the basic operations on the data produced during the measurements, namely import, storage, visualization and preprocessing. The first module responsible for data import loads curves from the native format created during measurement into the storage module, which can be described as a classic relational database. The visualization module draws each Brdicka curve as a classic XY graph, and this enables an arbitrary set of curves stored in the database to be selected for depiction in a single image, see Figure 7. This feature proved to be very helpful when comparing various sets of curves. The data preprocessing module deals with counting the requested wavelet transformation coefficients as well as all other values utilized throughout the paper (e.g., the minimum and maximum in a specified interval, etc.).

All pictures of curves in this paper were generated by our software tool, which is available from its author on request - please send an e-mail to vyslouzilova@labe.felk.cvut.cz. The original data is available from the first author of the paper on request.
